# Do Current Measures of Social Exclusion Depict the Multidimensional Challenges of Marginalized Urban Areas? Insights, Gaps and Future Research

**DOI:** 10.3390/ijerph18157993

**Published:** 2021-07-28

**Authors:** Rocío Vela-Jiménez, Antonio Sianes

**Affiliations:** Research Institute on Policies for Social Transformation, Universidad Loyola Andalucía, 41704 Seville, Spain; mrvela@uloyola.es

**Keywords:** social exclusion, multidimensional indicators, disadvantaged urban areas, social transformation, local development, inclusive and sustainable cities, social policy, 2030 Agenda for Sustainable Development

## Abstract

According to the United Nations, 70% of the world’s population will live in cities by 2050, increasing the proliferation of areas of social exclusion and thus polarization and segregation. The establishment of multidimensional measures seeks to identify such situations of social exclusion to inform social policies and interventions. However, some concerns emerge: Are these measures catching the needs of people living in particularly disadvantages areas? Do they offer a human-centred approach or a territorial focus? Is the multidimensionality of such measures reflecting nonmaterial aspects such as health, access to liveable environments or political participation? To analyse how the scientific literature is addressing the measurement of social exclusion to tackle such urban challenges, a systematic review following the PRISMA guidelines was performed in the Web of Science database. After screening following the inclusion criteria, 28 studies were identified that analysed systems of indicators that multidimensionally examined social exclusion at the individual and/or family level in urban contexts. Despite studies being eminently limited to some Western countries, the results revealed a broad diversity. However, very few of them fully focused on the specific characteristics of marginalized urban areas, and most found serious difficulties in overcoming a material approach.

## 1. Introduction

The concept of social exclusion emerged for the first time in Europe in the 1970s, in the context of an economic restructuring and concerns about the risks to social cohesion and stability [1]. Conventional measures of poverty and deprivation were considered inadequate to capture the alienation, isolation or “exclusion” of the social normative functioning that arose as a result of such economic insecurities [1]. Social exclusion implies not only the denial or lack of resources, goods and services, but also the inability to participate in normal relationships and activities available to most people in society, whether in the economic, social, cultural or political sphere [2]. In this way, it is defined as a complex and multidimensional process, not only a negative condition in itself, but a disturbing element for social and economic development, both individually and socially [3].

In a context in which the links between the economic and social spheres are becoming increasingly important [4], the eradication of social exclusion in urban contexts has become one of the greatest challenges at international level. In this line, the United Nations included, in the 2030 Agenda for Sustainable Development, the Sustainable Development Goal (SDG) 11, aimed at achieving sustainable cities and communities, with special attention to socially excluded neighbourhoods [5]. To achieve this goal, the measurement has become a central issue from both social and political points of view [6].

However, the measurement of social exclusion is still an open debate, as different proposals coexist, and there seems to be no consensus on theoretical and methodological approaches in empirical studies [3]. To shed light on this debate, this article will offer a systematic review of this body of literature: first, to identify its trends and evolution, as those aspects for which consensus has begun to emerge and those for which there is still an open debate; and second, to inquire whether such proposals are adapted for the measurement of social exclusion in the most disadvantaged urban contexts.

### 1.1. Social Exclusion as a Multidimensional Phenomenon

At the end of the 1970s, an academic trend led by pioneering authors such as Peter Townsend [7] aimed to introduce nonmonetary measures into the measurement of poverty, to catch the multidimensionality of social exclusion. According to these authors, people are socially excluded when they lack the resources to cover their basic needs, but also when they cannot access the living conditions and services that are common in the societies to which they belong. Their resources are so below those of the average individual or family that they are unable to fully participate in societal life [7].

Authors such as Mack and Lansley [8] delved into this idea, developing the consensual view of needs approach. Such approach defines social exclusion as the forced lack of socially perceived needs, thus distinguishing between those who lack needs by choice and those who cannot afford them [8]. According to this approach, the socially perceived needs that give access to decent living conditions are established by society itself and not in a generalized way by academic or political opinion [8]. As emphasis is placed not only on income threshold, but also on the forced lack of socially perceived needs, social exclusion implies not only poverty in the form of low income, but also educational disadvantage, poor health, inadequate housing or even living in poor areas [9].

In parallel, the approach to human capabilities proposed by Amartya Sen [10] provides a complementary lens for the study of poverty and social exclusion [11]. To understand these issues, Sen proposes taking into consideration the characteristics of the individuals and the context they inhabit. This contextualization allows evaluating to what extent these individuals have the capabilities to participate in key activities of the society in which they live under conditions of equality [9]. Building upon multidimensional and contextual approaches to exclusion, in the 1990s the United Nations recognized that income is not an end in itself, but a means to achieve a broader objective: human development [12]. To achieve the objective of human development, there are both economic and noneconomic factors, such as crime levels, the position of women and respect for human rights. With this in mind, the United Nations Development Programme (UNDP) has regularly published since 1992 a classification of countries in terms of their Human Development Index (HDI) [12].

Today, most international agendas embrace this multidimensional approach in the fight for social justice. In this line, the 17 Sustainable Development Goals (SDGs) proposed in the 2030 Agenda for Sustainable Development aspire to “transform our world” [5] by combining the three dimensions of sustainable development; i.e., economic, social and environmental, in an integrated manner. In the same vein, the EU is making efforts to develop policies that reduce the risk of poverty and social exclusion and reinforce social cohesion, materialized in the Laeken Indicators that, from a multidimensional approach, aim to measure poverty and social exclusion in EU member states.

In conclusion, the multidimensionality of social exclusion seems to have been the prevailing view since Atkinson [13] proposed approaching exclusion from a rights-based approach. This multidimensionality comprises three key ideas [14]: the relativity of exclusion, by which it must be taken into account in a given moment and context; the dynamics of exclusion, which refers to the need for understanding it over time and therefore to carry out long-term monitoring; and finally agency, which seeks to distinguish when the process by which people are excluded is by their own choice, or by the action of others or due to structural issues. Social exclusion understood using these key ideas reflects a multidimensional lack of connection, the elements of which are related not only to the characteristics of individuals, but also to communities and the social and physical environments in which people live [15]; thus the importance given by academic researchers to the measurement of social exclusion, including the conditions and dynamics of the territories [16].

### 1.2. The Debate Concerning the Measurement of Social Exclusion in the Urban Context

When studying social exclusion today, focusing the gaze on the urban environment is especially relevant. According to the United Nations, 55% of the world population currently lives in cities, estimating that by 2050 this figure will reach 70% [17]. This population growth in cities is increasing residential polarization and segregation, thus proliferating disadvantaged urban areas where social exclusion and marginalization is the norm and not the exception [18]. In absolute terms, it is estimated that of the nearly 4 billion people who lived in cities in 2015, 883 million people, representing almost 25%, lived in marginal neighbourhoods [19], a tendency that is only expected to increase.

The characteristics of the context in which an individual lives, not only at a given moment but also over time, have been deemed as essential when monitoring social exclusion [9,20]. The access to resources socially perceived as necessary is crucial to participate fully in society, and it has been one of the elements considered by literature to generate transformative processes towards a society in which everyone fits [1]. Thus, it is worth asking whether the metrics that address social exclusion in urban contexts are useful when focused on the socially excluded neighbourhoods that coexist in a segregated way in cities.

For decades, the scientific literature has made great efforts to provide metrics and measures that deal with the complexity of the concept of social exclusion. However, academic discussion regarding this issue continues [21]. One of the main debates in the field, which overcomes the goals of this review, focuses on the relation of territorial-based social exclusion and individual/household-centered social exclusion. This line of research tries to identify the linkages and differences of studying social exclusion in specific territories, and social exclusion as experienced by specific populations. Towards the goals of our study, it is worth focusing on the latter, where the main dispute probably revolves around which metrics should be used to represent the reality of those people who are excluded from society. Regarding how such measure is constructed, there is also a debate about the adequacy of developing an index that measures social exclusion in an aggregate manner [1]. This measure can leave out many important aspects involved in social inequality by merging the multidimensional complexity of social exclusion into a single index. Stewart [22] summarized this dilemma by stating that “different indicators tell very different stories” [1]. However, such aggregated measure is of unequivocal utility for decisions and policymakers, as they require the provision of evidence-based urban data, schemes and knowledge to make informed decisions [23].

Using the methodology and materials described in the following section, this systematic review aims to contribute to this debate. This review will identify how researchers have addressed, over time, the construction of dimensions and indicators that account for the multidimensionality of social exclusion. Then, by analysing and systematizing the different measures, it aims to elicit consensus, open debates, insights and gaps that can help provide guidelines for future research. It will contribute to better inform social policies able to generate transformative processes that dissolve the dynamics of social exclusion and thus favour, in local development, the conformation of inclusive and sustainable cities.

## 2. Materials and Methods

The present study is a systematic review of the scientific literature that has addressed the construction of metrics to measure social exclusion in a multidimensional way in urban contexts. To do so, the Preferred Reporting Items for Systematic Reviews and Meta-Analyses (PRISMA) methodology was implemented. The PRISMA Statement consists of a series of 27 steps and items to be considered, represented in a flux diagram that includes four phases: Identification, Screening, Eligibility and Included [24].

To identify the most relevant literature on the topic, the databases used for this search were the main collection of the Web of Science and Scopus. In recent years, most systematic reviews have focused on these repositories because they archive the most prominent academic forums of any discipline [25,26]. Given the objectives of the study, only the Social Sciences Citation Index (SSCI) of the Web of Science and the Social Science category of Scopus have been included, from 1956 to the present. Similarly, and to the extent that it was intended to identify only studies based on empirical evidence, a filter was used to retrieve only articles in the search results. Finally, the search language was English, the dominant language in academic research.

The search strategy intended to limit the sample to the objective of finding scientific articles that account for the multidimensionality of social exclusion, using terms referring to metrics, and specifically those that are related to social exclusion understood in a multidimensional way. Therefore, the following terms were used in the search vector: TS = (((“Indicators” OR “Measures” OR “Index” OR “Frameworks”) AND (“Social Inclusion” OR “Social Exclusion” OR “Excluded from Society” OR “Social Segregation”) AND (“Poverty” OR “Development” OR “Social Quality”))).

The inclusion and exclusion criteria used for screening articles, after reviewing the title and abstract, were determined with the intention of refining the search for scientific articles towards the intended sample: (a) articles that analyse systems of indicators to measure social exclusion in a multidimensional way, thus excluding approximations based on mere criteria of poverty or material deprivation; (b) studies that use metrics developed at individual or household level, as suggested by the literature [15] to measure social exclusion from the contexts and specific characteristics of individuals; (c) studies that refer to urban contexts, thus excluding those referring to rural areas; and (d) studies not limited to the analysis of the exclusion of specific groups, thus rejecting those articles that analyse only groups of individuals through specific characteristics such as age, ethnicity or origin.

These exclusion criteria were incorporated into the coding sheet of all the articles retrieved with the search strategy; cascade screening was performed, assigning a number to each exclusion criterion to identify the excluded articles.

Once selected, to extract and systematize the information from each contribution included in the review, a new coding sheet was designed, in which the information was systematized in four axes of information, as shown in Table 1.

The first axis refers to the identification of the contributions, including information about the title of the article and the authors, and the year and the journal of publication.

The second axis focuses on the application of the studies, with respect to the countries in which they were carried out, whether they focus on marginalized urban areas, the definition given to the reality they intend to measure and finally the moment of such measurement.

The third axis aims to systematize the information for the construction of the indicators system, such as the database and who carries out its application, whether the unit of analysis refers to households or individuals, the size of the sample and information on the theoretical and methodological approaches of each measure.

Fourth, the multidimensional system of indicators used in each article is thoroughly described, identifying whether the measurement is longitudinal or cross-sectional, the number and type of dimensions and indicators used, the percentage of objective and subjective indicators, whether such dimensions and indicators are aggregated in a single index, by what technique this aggregation is carried out and finally which dimensions and indicators are prioritized if done.

While some articles focus exclusively on the construction of a system of indicators to measure social exclusion, others use this metric to deepen further analyses. Due to systematization goals, the focus will be on the indicator systems, while relevant findings of the derived analyses will be incorporated in the discussion section.

## 3. Results

### 3.1. Identification of Studies

As a result of the search conducted in May 2020, 379 articles published from 1993 to 2019 were identified. After reading the title and abstract and applying the exclusion and inclusion criteria, 33 articles met the inclusion criteria (N = 33), thus 346 articles were eliminated.

The excluded articles were grouped into exclusion categories. A total of 135 articles were rejected as they did not analyse indicator systems, and 39 were rejected because their topics did not address social exclusion. A total of 65 articles were rejected because, while applying indicator systems, they did not apply a multidimensional analysis. Four more articles were discarded because they referred to rural contexts, and 43 articles were rejected because the multidimensional indicators they used were not applied at the individual or household level.

Finally, 65 articles were discarded because they referred to a specific group of individuals (ethnic groups, migrants, women, etc.).

**Figure 1:**
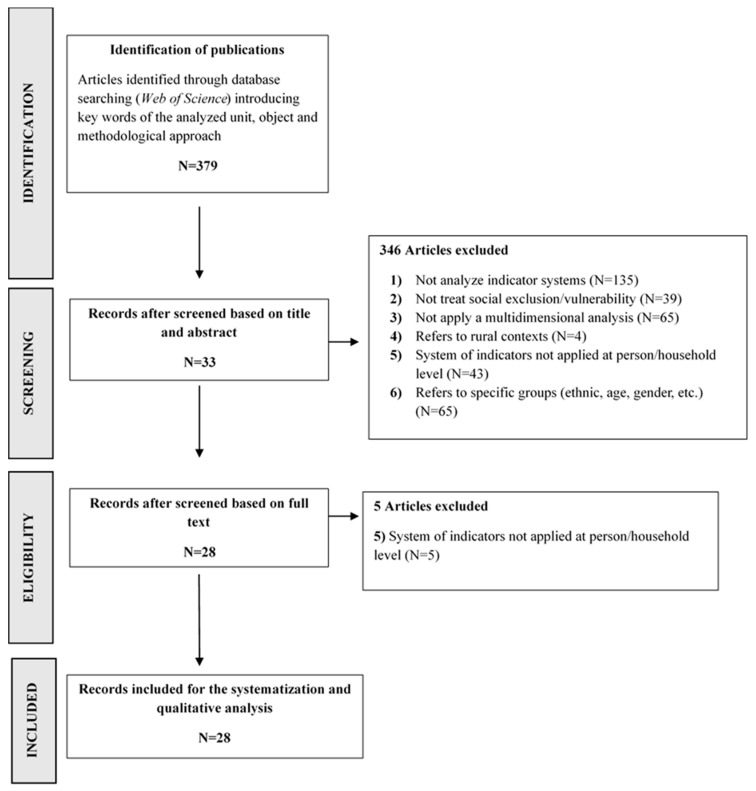
Flow diagram of study selection.

After critically reading each of the 33 articles that met the inclusion criteria, five more articles were discarded because they did not address the individual or household levels. Thus, the final sample of articles that met the inclusion criteria was 28 studies (N = 28). The selection process is summarized in Figure 1, following a PRISMA flow diagram [24].

Table 2 provides the list of the 28 research articles considered in the systematic review. Contributions were published from 1993 to 2019, but two-thirds of the papers were published recently, from 2010 to date, which highlights the topicality of the debate.

### 3.2. Application of the Studies

Although there were two studies that referred to the measurement of social exclusion in the European Union (EU) [3,6], most of the contributions were made at the national level. The studies were carried out mainly in countries in Europe, except for some conducted in countries such as Australia [15,38,39,44], the United States (US) [37] and Mexico [45]. Notably, all of the countries were members of the Organization for Economic Cooperation and Development (OECD), and considered to be highly developed according to the United Nations.

The United Kingdom (UK), with eight articles, and Ireland with six, were the countries with the greatest number of contributions. In studies conducted in such countries, the following authors stood out: Whelan, Nolan, Bradshaw, Callan, Burchardt, Maitre and Watson. Australia contributed four articles, where authors such as Boreham, Saunders and Scutella stood out. The Netherlands was another important context under study, with a total of three articles. This and subsequent information are systematized in Table 3.

Due to the selection criteria, all the studies included in this review were conducted in the urban context. However, only two of them [29,43] focused their analysis specifically on marginalized urban areas.

Regarding the moment of the analysis, the data used in the studies stemmed from different periods, from 1970 to 2013. The majority of studies (19) were concentrated in the period of 2004 to 2013. The relative age of the data, even in the most recent studies, shows an important delay in the data available for research in the field.

### 3.3. Construction of the Indicators Systems

The data, as well as the dimensions and indicators used in each study, mostly came from periodic surveys conducted in different countries, in some cases through specialized research centres or statistical offices. The proposed indicators systems were thus constructed with secondary information. The only exception was the study by Vrooman and Hoff [36], which obtained data directly through the application of qualitative and quantitative techniques, such as discussion groups, interviews and surveys conducted by the authors.

Of the 28 articles included in this systematic review, 22 established the unit of analysis at the individual level, while only six referred to households. In the latter case, the average sample size of households was 3914, with 10,000 households being the largest sample size [42], and 1300 households being the smallest sample size [30]. With respect to the sample used in the studies conducted at the individual level, the average was 21,176 individuals, with 212,674 being the largest sample [45], and 584 being the smallest sample [20].

With respect to the methodology used to construct a system of measurement of social exclusion in a multidimensional way, greater diversity was observed in the quantitative and qualitative techniques. Among them, we found cumulative counting-sum approaches; statistical techniques such as factorial analysis, latent class analysis, structural equations, self-organizing-maps, non-linear principal component analysis, etc.; and even bottom-up approaches based on information extracted from discussion groups, interviews or surveys.

### 3.4. Characterization of the Indicator Systems to Account for Social Exclusion

According to most of the studies included in this review, the combination of indicators to analyse the level of social exclusion faced by certain individuals could be a function of: (1) the number of dimensions in which exclusion is experienced, (2) the number of exclusion indicators present within each dimension, and (3) the time when these indicators are present for the individual [39].

Starting with the last aspect, more than half of the studies reviewed (17) were carried out at a certain time (cross-sectional), while only 11 monitored the situation and the evolution of exclusion longitudinally over time.

Regarding the dimensions of exclusion, the mean number of dimensions used was 5.5, and the largest number of dimensions used was 12 [43], the latter perhaps explained by the fact that it was a longitudinal study of deprivation from childhood to adulthood.

Regarding the type of dimensions used to measure social exclusion at the multidimensional level, significant variation was found among the studies. To facilitate synthesis, all were grouped into 10 base dimensions, combining those used in three of the most relevant studies [21,39,44]. These 10 dimensions were classified into two broader categories, as suggested by two of the contributions [36,40], according to whether they generated economic–structural exclusion or sociocultural exclusion. The economic–structural exclusion category thus included seven dimensions: income (which includes both income and employment); material deprivation (of goods and services); health and disability (including both mental health and physical, intellectual and sensory disabilities); education; housing; security; and access to basic services. Within the sociocultural exclusion category, we found three dimensions: community, social and political participation, and social support.

**Figure 2:**
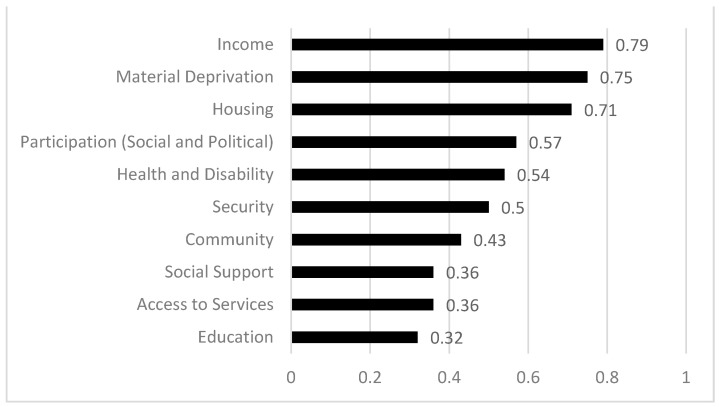
Prevalence of each dimension.

Figure 2 shows the percentage of studies that included each of the different dimensions in their multidimensional perspective. Some of the dimensions that compose economic–structural exclusion, such as income, material deprivation, housing, health and disability, were the most used. Among them, social and political participation emerged as a notable exception, a dimension belonging to sociocultural exclusion that also appears to be one of the most used.

Finally, regarding the number of indicators, 17.20 were used on average, the largest number of indicators being 55 [32], and the lowest eight [20].

With respect to the type of indicators used, it was again interesting to distinguish between objective and subjective indicators, in line with the existing debate in the literature. Within the studies analysed, the vast majority were objective indicators, representing 98.86% of the total. Although the number of subjective indicators was very low, more than half of the studies applied a combination of both types of indicators, but in a very unbalanced manner. Of the 19 studies that included both types of indicators, only one [3] included more subjective than objective indicators. It is worth noting that none of the articles contained subjective indicators in the dimensions of material deprivation and education.

Combining the three elements mentioned; i.e., temporal approach, dimensions and indicators, the studies provided interpretations about the multidimensional social exclusion experienced by the individuals or households under study. Most studies opted for proposing an index that, by adding the dimensions, allowed measuring the overall level of social exclusion; only eight of the 28 articles analysed did not use an aggregate measure to assess social exclusion.

Of the 20 studies that did use indices to measure social exclusion, most of them (11) equalized the indicators and the dimensions, with nine performing a weighting to calculate the aggregate index. Of these, only three expressly prioritized certain dimensions, including social participation [29], access to basic social rights [36] and health [37].

It is also worth noting that, of the 20 studies that developed an aggregate measure, only nine carried it out using information from a single index. The remaining 11 studies did so in combination with other measures, such as subjective measures or the poverty line, which is established as a threshold in most European countries at 60% of the median general income.

All this information is included in Table 3, which summarizes the most relevant aspects of the systematization process.

## 4. Discussion

This review aimed to synthesize the scientific literature regarding the measurement of social exclusion in urban areas in a multidimensional way. The first observation was that, despite being the subject of an open debate for more than four decades, there are still different ways of conceptualizing social exclusion, as well as positions on whether establishing a measure that allows identifying the people who are excluded within a given society. In this section, some trends will be identified that can provide guidance on how social exclusion could be measured to inform social policies and interventions aimed at eradicating it. These trends are introduced in five points: the first one focuses on the localization of the research studies; the second on their territorial focus on disadvantaged urban areas; the third one deepens the nature and characteristics of data; the fourth addresses how each social exclusion measure is constructed, and thus is divided into three subtopics: its dimensions, its indicators and the time of coverage; and finally, once each measure has been scrutinized, the fifth point focuses on the pertinence of using an aggregate metric.

The first element worthy of analysis is that most of the literature identified focused on the context of European countries or in other OECD member countries. In fact, there was a slight primacy of studies focused on countries in the EU, which was to some extent surprising, as countries as the United States face several difficulties in social integration due to international migration and profound ethnical segregation. However, what amazed us the most was the absolute lack of research studies focused on developing countries. Thus, the first question that arose was to discern whether social exclusion, understood in a multidimensional way, is a problem that is conceptually used when trying to measure inequalities in the urban environment of Western societies (Globalized North). It seems as if, constrained by the traditional view of “development”, this concept emerges as a substitute for poverty, a term relegated to the study of developing countries where development cooperation circumscribes their action (Globalized South) [48]. It would be advisable to carry out a dialogue concerning the studies on social exclusion conducted in rich countries with those on multidimensional poverty conducted in impoverished countries. A bibliometric analysis would serve as an initial approach to see the extent to which these two areas are connected. Knowing this, one could inquire whether this apparent conceptual distinction could be overcome by cross-learning, leading to a more comprehensive discourse around the conceptualization and measurement of social inequalities in the world.

A second element that stands out is that despite having limited the selection to studies carried out in urban contexts, only a couple targeted those urban areas considered especially disadvantaged or marginalized [29,43]. The world in which we live is increasingly urbanizing, and urban areas where a large part of the population is in a situation of social exclusion are proliferating due to the social segregation process [18]. An important gap was found in studies that address this reality, which is only being aggravated by the COVID-19 crisis. In such contexts, the achievement of SDG 11 becomes a priority that depends, to a large extent, on the capacity to investigate and monitor the situation faced by this important part of the most vulnerable population. There is, therefore, a need to complement existing studies with others that provide a microanalysis of the situation of social exclusion in these disadvantaged urban areas.

Third, it is important to reflect on the data used by the articles analysed to measure social exclusion. Even the most recent studies use data that do not go beyond 2013, which warns of an overly long period lacking available data. Furthermore, the data came mostly from secondary databases. These data reported general situations of the society to which they referred, making it difficult to access specific data if one wants to focus on the situation of social exclusion faced by certain individuals, especially those living in marginalized urban areas. The combination of both elements shows an important gap with respect to studies on social exclusion in urban contexts. On the one hand, as seen during the 2008 crisis and the current COVID-19 crisis, some events generate immediate impacts regarding the exclusion of millions of people [49]. Generating knowledge with an average offset of approximately 5 years cannot inform effective policies that respond to the challenges stemming from such events. Shocks such as those mentioned lead to syndemics [50], impacting with greater force both marginalized urban areas and the people who inhabit them. It is therefore necessary to conduct studies with updated data collected at the microlevel and focused on these territories [51]. It seems useful to implement social observatories that address, from specific realities, the causes, dynamics and evolution of social exclusion to inform localized policies aimed at improving the living conditions of the most vulnerable populations of our societies.

Fourth, and probably the most relevant aspect still open to debate, is determining how to effectively measure social exclusion. As reviewed by Scutella, Wilkins and Kostenko [39], there is an emerging consensus on the three aspects that should be considered: (1) the dimensions in which exclusion is experienced, (2) the indicators of exclusion present within each dimension, and (3) the time at which these indicators are present for an individual. However, open debates remain in each one of these aspects.

Starting with the latter, although most authors agree in their theoretical frameworks on the importance of considering a longitudinal approach to analyse the dynamics of social exclusion, more than half of the empirical articles were cross-sectional. The longitudinal structure of the data allowed examining the persistence of social exclusion, observing not only a snapshot of the circumstances of individuals, but also following their fate over time. Therefore, there is a need to conduct more longitudinal studies that provide evaluative information so that policies respond to the different challenges that occur in each phase of these situations of inequality [9].

Regarding the dimensions and indicators used to measure social exclusion, a first look could suggest that there is high variance among those employed in studies. However, the classification explained in the Results section shows how a certain consensus emerges about what individuals (households and their members) must have to be included in societies. Thus, most proposals refer to access to material resources (including housing); income to afford these material resources and to carry out nonmaterial activities (such as vacations and socialization); access to employment (in part as a means of generating income); access to education and health care; absence of discrimination (including victimization or lack of personal and community security); opportunities for social participation (including removing any obstacles to such participation); and opportunities to translate their demands into political options. However, when deepening the analysis of the dimensions, it was observed how the literature gave a differential weight to those that accounted for economic–structural exclusion, considering to a lesser extent those that define sociocultural exclusion.

In this regard, an existing debate regarding the dimensions of education and employment must be noticed. It is not yet solved in the literature whether lack of education and employment should be considered dimensions of exclusion or if, on the contrary, they should be studied as part of the socioeconomic characteristics of the individual, as they condition their ability to be included in society. This dilemma calls for future research that, using structural equation models and the like, contributes to determining the role of education and employment in the situation of social exclusion of individuals, and their potential to generate processes of social inclusion.

The third and final aspect of the measurement of social exclusion refers to the indicators. The existing debate in the literature regarding the role that qualitative approaches could play in the measurement of social exclusion has no translation in empirical studies. Regarding the use of objective versus subjective indicators, the literature mostly opted for the former. Although many studies made explicit mention of the need to build a multidimensional account of social exclusion through the use of mixed approaches [46], the use of qualitative techniques in the scientific literature analysed was residual. This gap in the literature opens lines of research in two ways: the role that perceptions play in the processes of social exclusion, and the contributions that can be made to the construction of multidimensional metrics by the use of qualitative methodologies such as focus groups, interviews and participant observation.

The fifth and final element of discussion is another of the most relevant debates in academia: the pertinence of using an aggregate metric of exclusion through a single index or, on the contrary, whether a better account of multidimensionality is provided by presenting the dimensions separately. Within the literature analysed, there was a tendency to aggregate measures into a single metric, which allowed the identification of whether individuals were in a situation of social exclusion. To enrich the multidimensional view, most articles used this metric combined with other measures, so that more variables were considered in addition to those used in their own index. Among the arguments for building a single index is that its creation can facilitate the monitoring of processes and situations of social exclusion to facilitate the information of policies [52]. For their part, the detractors of this option point out that the experience of social exclusion is multifaceted and complex, and that ethically relevant information regarding the advantages and disadvantages accumulated in the multiple dimensions is lost in aggregation [47].

Given the breadth demonstrated in this review of the concept of social exclusion, it seems that a single indicator is not sufficient to describe the different situations of social exclusion [3]. It may be more appropriate to combine an aggregate index with analyses of each of dimension to complement the information that can be lost in aggregation. In any case, this debate can lead to future investigations that will cover the existing gaps and contribute to the scientific literature with new perspectives to move towards a measurement that accounts for the complexity of the multidimensionality of social exclusion while being useful for monitoring and decision making.

Finally, remarkable differences were detected when using methodologies and techniques to determine how the different dimensions should contribute to the measurement of social exclusion. The scientific literature showed that the use of thresholds to distinguish excluded people from those not excluded is a difficult task, in part due to the multidimensional nature of exclusion. Both the selection of the indicators and the threshold points used were often arbitrarily established, a weakness recognized by researchers themselves. There were still a few cases in which thresholds were constructed based on the perceptions of the main subjects instead of starting from the judgement of the researchers. Once again, in this regard, an important line of future research is opened that merges the suggestions made by academia with the perceptions of the subjects who are immersed in effective situations of social exclusion.

## 5. Conclusions

This review has revealed the complexity of establishing a multidimensional measure to monitor the evolution of social exclusion in our societies. Several gaps to be addressed in the scientific literature have been identified, as well as certain key lines of research so that future research can unravel the complexity of measuring social exclusion. This is especially important in a globalized society in which the population is trending upward in cities, along with an increase in social segregation, which is particularly exacerbated by the current crisis caused by the COVID-19 pandemic. Generating knowledge in this regard will be essential to inform social policies and interventions that effectively combat poverty and social exclusion in cities. This need for accurate, localized and timely information and data also calls for the pertinence of supporting and developing national statistical offices in countries in the Global South, as their lack could be one of the reasons of being widely underrepresented in the academic literature.

The main weakness identified in the academic literature on this topic was that top-down approaches to measure social exclusion overwhelmingly prevailed. It is necessary to complement these studies with a bottom-up strategy able to collect experiences of social exclusion and inclusion in specific contexts and from their protagonists. A call to incorporate participatory approaches to measure social exclusion can be found in the literature, but this trend is still a minority. Participatory action research (PAR) approaches can be particularly useful to provide a greater understanding of the perceptions and experiences of groups that are socially excluded. This will not only allow a better identification of excluded people, the nuanced characteristics of their exclusion and the evolution of their situation over time, but also will offer clues to inform effective social policies and interventions that generate processes of social inclusion. These policies will be essential to achieve the objectives of SDG 11 and other international strategies addressing urban sustainability, especially in marginalized areas.

Apart from the future lines of research identified in the Discussion section, it is worth noting a last one related to the main limitation of this study. Having focused the analysis on the academic contributions published in indexed journals, it may be important to investigate the grey literature, as some of the main weaknesses detected may already be in the process of being addressed. It is possible that knowledge is being generated at the local level with participatory approaches, transforming the way in which social exclusion is studied. If this is the case, it would be necessary to systematize their learning to share knowledge through the main forums of academic debate, and thus effectively contribute to the enhancement of inclusive and sustainable cities based on the struggle for human dignity and social justice.

## Figures and Tables

**Table 1:** Axes of systematization for the systematic review.

Identification of Studies	Application of Studies	Construction of the System of Indicators	Characteristics of the System of Indicators
Title	Country/Countries	Database	Temporal approach
Author	Focus on marginalized urban areas	Unit of analysis	Number of dimensions
Journal	Social reality measured	Sample	Type and nature of the dimensions
Year	Moment of the measurement	Methodological approach	Number of indicators
			Subjective indicators
			Aggregation
			Prioritized dimensions

**Table 2:** Articles included in the review.

Reference	Title	Journal
[27]	Resources, Deprivation and the Measurement of Poverty	Journal of Social Policy
[28]	Social Exclusion in Britain 1991–1995	Social Policy & Administration
[29]	Social indicators and living conditions in the Netherlands	Social Indicators Research
[30]	Overlaps in dimensions of poverty	Journal of Social Policy
[12]	Equity-sensitive indicators of living standards with an application to Northern Ireland	Scottish Journal of Political Economy
[15]	Towards new indicators of disadvantage: Deprivation and social exclusion in Australia	European Societies
[31]	Measuring material deprivation with EU-SILC: Lessons from the Irish survey	International Journal of Social Welfare
[32]	Poverty, welfare problems and social exclusion	Australian Journal of Social Issues
[6]	Using Non-Monetary Deprivation Indicators to Analyze Poverty and Social Exclusion: Lessons from Europe?	Journal of Policy Analysis and Management
[33]	Mapping patterns of multiple deprivation using self-organising maps: An application to EU-SILC data for Ireland	Social Science Research
[34]	Understanding the socio-economic distribution of multiple deprivation: An application of self-organising maps	Research in Social Stratification and Mobility
[3]	Evaluating contemporary social exclusion in Europe: a hierarchical latent class approach	Quality & Quantity
[35]	Deriving Multidimensional Poverty Indicators: Methodological Issues and an Empirical Analysis for Italy	Social Indicators Research
[36]	The Disadvantaged Among the Dutch: A Survey Approach to the Multidimensional Measurement of Social Exclusion	Social Indicators Research
[37]	The Counting-Based Measurement of Multidimensional Poverty: The Focus on Economic Resources, Inner Capabilities, and Relational Resources in the United States	Social Indicators Research
[38]	An alternative measure of social wellbeing: analysing the key conceptual and statistical components of quality of life	Australian Journal of Social Issues
[39]	Intensity and persistence of individuals’ social exclusion in Australia	Australian Journal of Social Issues
[40]	The Socially Excluded in the Netherlands: The Development of an Overall Index	Social Indicators Research
[41]	Comparative Assessment of Methods for Measuring Consensual Poverty: Sort Card Versus CAPI	Social Indicators Research
[42]	What Have We Called as “Poverty”? A Multidimensional and Longitudinal Perspective	Social Indicators Research
[43]	From Childhood Deprivation to Adult Social Exclusion: Evidence from the 1970 British Cohort Study	Social Indicators Research
[44]	Social inclusion, exclusion, and well-being in Australia: meaning and measurement	Australian Journal of Social Issues
[11]	Material poverty and multiple deprivation in Britain: the distinctiveness of multidimensional assessment	Journal of Public Policy
[45]	Multiple Deprivation, Severity and Latent Sub-Groups: Advantages of Factor Mixture Modelling for Analysing Material Deprivation	Social Indicators Research
[46]	Non-Monetary Indicators and Multiple Dimensions: The ESRI Approach to Poverty Measurement	Economic and Social Review
[20]	The Portuguese version of the European Deprivation Index: Development and association with all-cause mortality	Plos One
[47]	Measuring Well-being: A Multidimensional Index Integrating Subjective Well-being and Preferences	Journal of Human Development and Capabilities
[21]	From Income Poverty to Multidimensional Quality of Life	Economic and Social Review

**Table 3:** Systematization of the multidimensional measures of social exclusion.

Reference	Country	Dimensions	Indicators	Aggregated Index	Weighting	Database	Approach
[27]	Ireland	4	25	Yes	Nonweighted	Large-Scale Household Survey	Cross-sectional
[28]	UK	5	11	No		British Household Panel Survey (BHPS)	Longitudinal
[29]	Netherlands	8	26	Yes	Weighted	Netherlands Permanent Quality of Life Survey (POLS)	Longitudinal
[30]	UK	3	40	No		Poverty and Social Exclusion—Britain (PSE)	Cross-sectional
[12]	UK	2	42	Yes	Nonweighted	Poverty and Social Exclusion—Northern Ireland (PSENI)	Cross-sectional
[15]	Ireland	5	46	No		European Community Household Panel (ECHP)	Cross Sectional
[31]	Sweden	3	55	Yes	Weighted	European Statistics on Income and Living Conditions (EU-SILC)	Cross-sectional
[32]	Australia	4	53	Yes	Nonweighted	Community Understanding of Poverty and Social Exclusion Survey (CUPSE)	Cross-sectional
[6]	EU	6	25	Yes	Nonweighted	EU-SILC	Longitudinal
[33]	Ireland	5	44	No		EU-SILC	Cross-sectional
[34]	Ireland	5	44	No		EU-SILC	Cross-sectional
[3]	EU	3	16	No		Eurobarometer	Cross-sectional
[35]	Italy	5	44	Yes	Weighted	EU-SILC	Cross-sectional
[36]	Netherlands	4	15	Yes	Weighted	Own survey by authors	Cross-sectional
[37]	US	3	9	Yes	Weighted	General Social Survey (GSS)	Cross-sectional
[38]	Australia	6	25	Yes	Weighted	Survey of Queensland Households (SQH)	Longitudinal
[39]	Australia	7	29	Yes	Nonweighted	Household, Income and Labour Dynamics in Australia (HILDA)	Longitudinal
[40]	Netherlands	4	42	Yes	Nonweighted	EU-SILC	Cross-sectional
[41]	UK	8	46	No		Omnibus Survey	Cross-sectional
[42]	UK	6	34	No		BHPS	Longitudinal
[43]	UK	12	43	Yes	Nonweighted	British Cohort Study (BCS70)	Longitudinal
[44]	Australia	11	48	Yes	Nonweighted	Framework Australian Government	Longitudinal
[11]	UK	9	29	Yes	Weighted	BHPS	Cross-sectional
[45]	Mexico	6	10	Yes	Nonweighted	National Survey of Household Income and Expenditure (ENIGH)	Cross-sectional
[46]	Ireland	4	12	Yes	Nonweighted	Living in Ireland Survey (LII)	Longitudinal
[20]	Portugal	2	8	Yes	Weighted	EU-SILC	Longitudinal
[47]	UK	3	21	Yes	Weighted	BHPS	Longitudinal
[21]	Ireland	11	35	Yes	Nonweighted	EU-SILC	Cross-sectional

## Data Availability

No new data were created or analyzed in this study. Data sharing is not applicable to this article.
